# Tracheal Diverticula in People with Cystic Fibrosis on Elexacaftor/Tezacaftor/Ivacaftor: An Italian Multicenter Retrospective Study

**DOI:** 10.3390/jcm14072320

**Published:** 2025-03-28

**Authors:** Laura Venditto, Antonella Tosco, Angela Sepe, Alice Castaldo, Chiara Cimbalo, Cristina Fevola, Marco Di Maurizio, Roberto Baggi, Stefano Avenali, Vito Terlizzi

**Affiliations:** 1 Cystic Fibrosis Center, Azienda Ospedaliera Universitaria Integrata, 37126 Verona, Italy; 2 Respiratory Endoscopy Unit, Meyer Children’s Hospital IRCCS, 50139 Florence, Italy; 3 Cystic Fibrosis Regional Center, Paediatric Unit, Department of Maternal and Child Health, A.O.U. Federico II, 80131 Naples, Italy; 4 Dipartimento di Sanità Pubblica, Università Federico II, 80131 Naples, Italy; 5 SC di Pneumologia e UTSIR AORN Santobono Pausillipon, 80131 Naples, Italy; 6 Dipartimento di Scienze Mediche Traslazionali, Sezione di Pediatria, Centro di Riferimento Regionale Pediatrico FC Campania, Università di Napoli Federico II, 80131 Naples, Italy; 7 Cystic Fibrosis Regional Reference Centre, Department of Paediatric Medicine, Meyer Children’s Hospital IRCCS, 50139 Florence, Italy; 8 Department of Radiology, Meyer Children’s Hospital IRCCS, 50139 Florence, Italy

**Keywords:** cystic fibrosis, elexacaftor/tezacaftor/ivacaftor, tracheal diverticulum, bronchoscopy

## Abstract

**Background/Objectives**: Cystic Fibrosis (CF) is an autosomal recessive genetic disorder caused by variants in the gene encoding the cystic fibrosis transmembrane conductance regulator (CFTR) protein. Recently, a targeted therapy for CF has been developed, represented by the CFTR modulators that enhance or restore the function of the CFTR protein. The most recent is the combination of three modulators, Elexacaftor, Tezacaftor, and Ivacaftor (ETI). This study describes the presentation, management, and follow-up of tracheal diverticulum (TD) in pwCF receiving ETI therapy. **Methods**: This retrospective study included people with CF (pwCF) on ETI treatment and followed up in two CF Italian centers who developed an asymptomatic TD, diagnosed incidentally at chest CT scan. **Results**: Among 268 pwCF receiving ETI, three (1.19%) were diagnosed with TD identified after chest CT and were included in this study. Endoscopic confirmation was obtained in one patient. All patients were on inhaled colistimethate, two of them for chronic *Pseudomonas aeruginosa* colonization, and one undergoing eradication therapy. **Conclusions**: TD may be identified in chest CT obtained in pwCF in treatment with ETI. Further studies and a longer follow up are needed to confirm these findings.

## 1. Introduction

Cystic Fibrosis (CF) is an autosomal recessive genetic disorder caused by variants in the gene encoding the cystic fibrosis transmembrane conductance regulator (CFTR) protein. This results in viscous secretions affecting the upper and lower airways [1,2] leading to chronic lung disease.

The phenotype of CF is characterized by elevated sweat chloride levels, exocrine pancreatic insufficiency (PI), progressive lung disease with chronic bacterial infections of lower airways, impaired growth, hepatobiliary manifestations, and male infertility [1,2].

The clearance of airways secretions has historically been considered crucial in the treatment of CF. It facilitates the movement of mucus and its expectoration, keeping the lungs as clear as possible. More severe lung disease is associated with worse nutritional status, respiratory infection caused by bacteria such as *Staphylococcus aureus* or *Pseudomonas aeruginosa,* and lung function abnormalities [2]. Thus, preventing lung damage and slowing its evolution has always been a priority in the treatment of CF. This explains the need to start respiratory treatment as soon as possible to facilitate the clearance of airway secretions and eradicate pathogenic microorganisms [2,3,4].

To date, the development of therapies that act directly on the genetic defect has significantly changed life expectancy and the quality of life in people with CF (pwCF). These drugs enhance or restore CFTR function based on the specific variant [5] and can be divided into two classes of small molecules: potentiators and correctors. The first class of drugs to be successfully developed was CFTR potentiators, such as ivacaftor (VX-770, IVA), which are small molecules that interact with the mutant channel to augment its opening probability, enhancing anion flux across the plasma membrane [6,7]. CFTR correctors, such as lumacaftor (VX-809, LUM), are pharmacological compounds that partially rescue the processing and trafficking defects of the misfolded CFTR protein to enable the protein to reach the cell surface [8]. The combination LUM/IVA (Orkambi^®^) is now approved in the USA and EU for patients who are homozygous for the F508del variant and who are 1 year of age and older.

The triple combination of Elexacaftor, Tezacaftor, and Ivacaftor (ETI) was approved in most European countries for people with CF (pwCF) aged ≥2 or 6 years, with at least one F508del, and in the USA it was also approved for pwCF with 177 other responsive *CFTR* variants [2,9,10]. This therapy has led to an improvement in clinical symptoms, lung function and morphology, pulmonary exacerbation rate, body weight, and quality of life [11,12,13].

While the primary focus of CF management has historically been on lung infection and the prevention of lung function decline, in the last decade there has been increasing awareness of upper airway involvement in CF pathology, such as tracheal diverticula (TD). TD are herniations of the tracheal mucosa that usually occur along the right posterolateral tracheal wall at the thoracic inlet between the cartilaginous and muscular portions of the tracheal wall. They can communicate with the tracheal lumen or appear as an isolated paratracheal air cyst [14].

TD can be congenital, due to an embryonal defect during the endodermal differentiation of the membranous posterior tracheal wall or the development of the cartilage rings [10]. They contain all tracheal layers and are usually filled with mucus [15]. An association with other congenital conditions, such as esophageal atresia, has also been described [16].

TD can also be acquired, usually caused by an increased intraluminal pressure [17] as in chronic cough, which leads to the herniation of the mucosal membrane through a weak point of the tracheal wall [17]. Acquired TD tends to be larger than congenital TD, with a wider opening, and usually arises from the posterolateral wall [17], possibly due to anatomical factors [18]. It is usually located though the trachealis muscle or laterally between the cartilaginous rings, and mostly from the right side because of the positional support of the left posterolateral esophageal wall [19].

Although the etiology is currently unknown, several causes have been described, such as trauma, infections, and high-pressure injuries [19], and it is speculated that some conditions associated with chronic cough, such as chronic obstructive pulmonary disease [20] and CF [21], could lead indeed to TD [22].

Clinically, most cases of TD are asymptomatic. The symptoms described include cough, especially during sleep due to retention of secretions that overflow the trachea [19], recurrent chest infections, and neck abscesses (since the TD can also act as a reservoir) globus sensation [23], neck pain, hemoptysis, and dysphonia because of direct pressure on the recurrent laryngeal nerve [24].

Limited data on TD in pwCF are available, with reported prevalence by chest computerized tomography (CT) scan of 28% (26/92) [25], 17.7% (20/113) [22], and 38% (35/93) in a CF cohort with end-stage lung disease requiring lung transplantation [26]. The prevalence reported in the general population is lower (2.38–8.1%) [27,28,29].

The clinical implications of TD in pwCF are not yet well defined. Conflicting studies exist regarding the correlation between the presence of TD and radiological lung damage [21,22]. Some studies have observed a worsening of lung function in pwCF with TD, although this correlation was not always statically significant [21]. This has led to the hypothesis that pwCF with TD may be exposed to a higher degree of airway inflammation [26].

This case series aims to describe the presentation, management, and follow-up of three pwCF in treatment with ETI, in whom TD were incidentally identified on follow-up chest CT.

## 2. Materials and Methods

Ethical approval for this retrospective case series was granted by the Ethics Committee of the Meyer Children’s Hospital IRCCS Florence, Italy, on 12 November 2024. For case series we don’t have an approval code, according to Italian laws.

### 2.1. Patients

This retrospective study included two Italian CF centers in Florence and Naples. The CF Center of Florence follows approximately 400 patients, both pediatric and adult patients, while the CF Center of Naples follows approximately 180 pediatric patients, both according to recent standards of care [30]. CF diagnosis was based on criteria according to Farrell et al. [31].

PwCF receiving ETI treatment who had a TD identified on chest CT were included in this study.

All patients treated with ETI underwent CT scan before the beginning of therapy and at least once during treatment.

All participants and/or their parents provided written informed consent for the anonymous use of data.

Patient demographical data and medical information, such as lung function, medical treatments, and microbiological surveillance, were retrieved from the medical records.

Spirometry was performed according to American Thoracic Society (ATS)/European Respiratory Society (ERS) guidelines [32].

Pancreatic insufficiency (PI) was defined based on at least two values of fecal pancreatic elastase lower than 200/g measured in the absence of acute gastrointestinal diseases [33].

*Pseudomonas aeruginosa* (Pa) chronic infection was defined using the modified Leeds criteria [34]. Pulmonary exacerbations were defined according to the CF Foundation’s criteria [35].

### 2.2. Imaging

CT images from patients in which the TD had been identified were reviewed by an independent radiologist, evaluating the site, size, number of TD, and searching for a communication with the trachea. Therefore, a comparison with previous imaging has been performed.

### 2.3. Bronchoscopy

Bronchoscopes were performed at the Respiratory Endoscopy Unit, A. Meyer Children’s Hospital (Florence, Italy), using flexible (Karl Storz^®^, Tuttlingen, Germany; Ø 2.5/3.7/5.2 mm) and rigid (Karl Storz^®^, Germany; Ø 3.0/3.5/4/4.5/5), looking specifically for orifices of the TD already detected at the previous chest CT. Endoscopic evaluations were performed under general anesthesia (sevoflurane inhalation and intravenous propofol), using facial mask ventilation during the flexible bronchoscopy and spontaneous assisted ventilation during the rigid bronchoscopy through a bag attached via flexible tubing to the ventilation port of the bronchoscope [36], ensuring a proper positive pressure to allow the visualization of the TD.

## 3. Results

Among 268 pwCF receiving ETI treatment and followed at the CF centers of Florence (mean age 27.3 years, range 6.3–61.4) and Naples (mean age 11.1 years, range 6–17.3), three (1.19%) were identified as having TD after undergoing a chest CT performed during treatment, and were included in this study.

The main features of these cases are reported in Table 1.

### 3.1. Patient 1

A 30-year-old male was diagnosed with CF for positive newborn screening (sweat chloride (SC): 93–99 mEq/L, CFTR genetic profile: F508del/E585X), according to the protocol used in the Tuscany region, Italy [37,38].

The clinical presentation included pancreatic insufficiency (fecal elastase < 50 µg/gr) and diffuse bronchiectasis predominantly in the upper pulmonary lobes, complicated by recurrent spontaneous left pneumothorax treated with talc pleurodesis. He was colonized by methicillin-resistant *Staphylococcus aureus* and chronic *Pseudomonas aeruginosa*, according to the Leeds criteria [34], in treatment with inhaled colistimethate.

According to Italian legislative guidelines, he started ETI at the age of 29 years due to the presence of at least one F508del allele. This resulted in improved lung function (FEV_1_ before starting ETI 56%, best 78% in the past year), and a reduced frequency of pulmonary exacerbations requiring antibiotics (less than once per year compared to four exacerbations annually before treatment).

**Figure 1:**
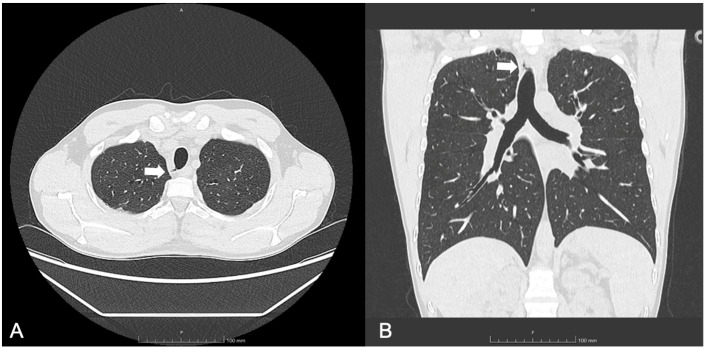
(**A**) Axial and (**B**) coronal lung windows at chest CT showing the tracheal diverticulum at the right posterolateral wall of the trachea in a cystic fibrosis patient (Patient 1). White arrows show the tracheal diverticulum.

**Figure 2:**
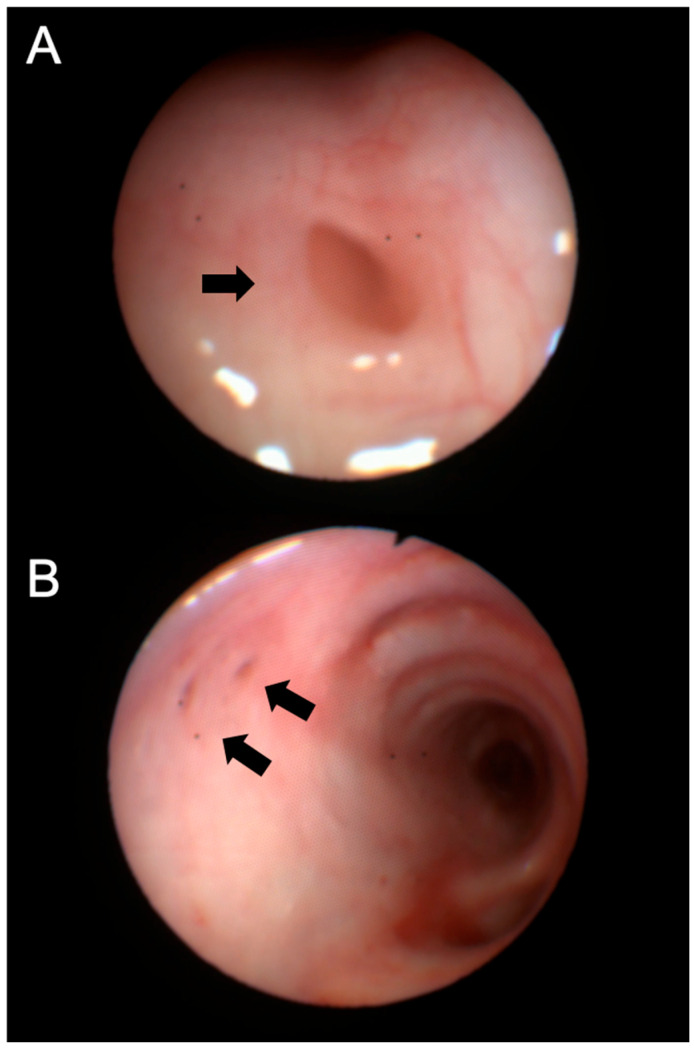
Bronchoscopy images of the tracheal diverticula identified in Patient 1, located at the right posterolateral wall (**A**) and the right paracarinal area (**B**). Black arrows show the orifice of the tracheal diverticulum into the tracheal lumen.

A follow-up chest CT, performed one year after initiating ETI therapy, revealed a new air-containing lesion located at the right posterolateral tracheal wall, communicating with the trachea (Figure 1). This lesion was not detected in the CT performed three years prior. The bronchoscopy confirmed the presence of a small TD in the right posterolateral wall at the mid-tracheal level (Figure 2A). As the patient remained asymptomatic, with stable FEV_1_ values, and no signs attributable to the TD, a watchful waiting approach was adopted given the TD’s small size. At the 6 months follow-up bronchoscopy, the TD was stable and clear of mucus, but a further TD was identified in the right paracarinal region (Figure 2B). An annual follow-up has been scheduled.

### 3.2. Patient 2

A 13-year-old girl with CF and pancreatic insufficiency (SC 125–125 mEq/L, CFTR genetic profile: F508del/Deletion 22–24; false-negative at CF newborn screening (NBS)) [39], was diagnosed at three years of age during hospitalization for complicated pneumonia. Her medical history included chronic cough, recurrent respiratory infections, and poor growth.

Before starting ETI, she presented with severe lung disease (FEV_1_ 45%) characterized by diffuse bronchiectasis and chronic colonization by methicillin-sensitive *S. aureus* and *P. aeruginosa* [34] in treatment with inhaled colistimethate. She had also been previously diagnosed with allergic bronchopulmonary aspergillosis and treated accordingly [40].

**Figure 3:**
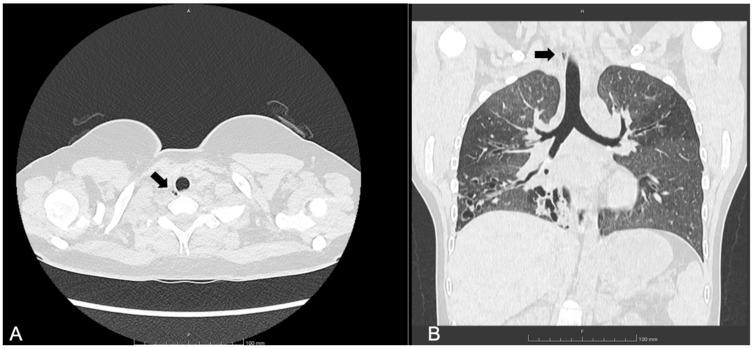
(**A**) Axial and (**B**) coronal lung windows at chest CT showing the tracheal diverticulum at the right posterolateral wall of the trachea in a cystic fibrosis patient (Patient 2). Black arrows show the tracheal diverticulum.

She started ETI at the age of 12. Following treatment, her lung function improved (best FEV_1_ in year after ETI initiation: 70%), and she experienced two pulmonary exacerbations requiring antibiotics in that year (compared to five pulmonary exacerbations annually prior to treatment). A follow-up chest CT scan performed one year after starting ETI revealed the presence of a paratracheal TD located at the junction of the mid and lower trachea (Figure 3), which was not detected on a CT scan five years prior. The patient remained asymptomatic, with no decline in FEV_1_ values.

A subsequent bronchoscopy was then performed, which did not identify the TD opening. After six months, a chest MRI confirmed the resolution of the TD.

### 3.3. Patient 3

A 10-year-old girl with CF and pancreatic insufficiency was diagnosed via NBS (SC 97–103 mEq/L, CFTR genetic profile: F508del/F508del) according to the protocol used in Campania region, Italy [41]. Prior to initiating ETI, her lung function was excellent (FEV_1_ 123%), although bronchiectasis was noted in the right middle lobe, in the lingula, and in the right upper lobe.

**Figure 4:**
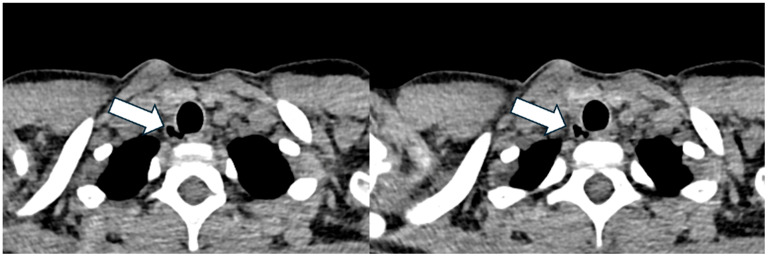
Axial lung window at chest CT showing the tracheal diverticulum at the right posterolateral wall of the trachea in a cystic fibrosis patient (Patient 3). White arrow shows the tracheal diverticulum.

She started ETI at the age of nine, following two years and six months of treatment with Lumacaftor/Ivacaftor. A CT scan performed one year after starting ETI, in the presence of *P. aeruginosa* isolation, revealed a 9 mm of maximal diameter TD (Figure 4), located at the posterolateral wall of the trachea at the T1 vertebral level. This TD was not present on the CT performed thirty months prior. The patient remained asymptomatic, with no decline in FEV_1_ values.

**Figure 5:**
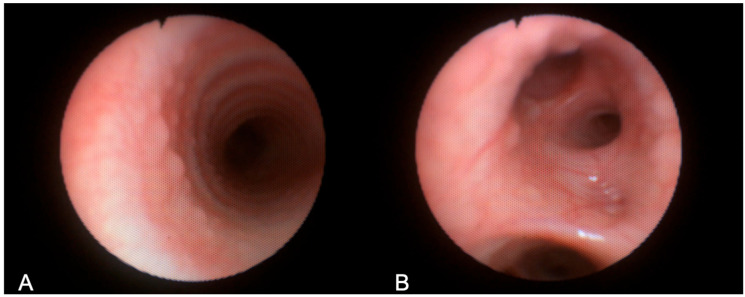
Bronchoscopy images obtained in Patient 3, showing mucosal granuloma in the tracheal wall (**A**) and bronchial walls (**B**).

A subsequent bronchoscopy using both flexible and rigid endoscopes did not confirm the presence of the TD. However, it revealed a nodule on the right vocal cord and small, fine granules that extended from the upper trachea to the main bronchi, suggestive of chronic airway inflammation. Minimal bronchial secretions were observed (Figure 5). A radiological follow-up one year after the bronchoscopy has been scheduled.

## 4. Discussion

We report three cases of TD incidentally detected in asymptomatic pwCF receiving ETI therapy. Endoscopic confirmation was obtained in only one case, highlighting, as CT scan is the gold standard to detect TD.

The lower frequency of TD observed in our cohort compared to previous studies [22,25,26] may be due to the lower median age of our patients and their better pulmonary function. Also, from a retrospective study [25], pwCF with TD had a lower initial FEV_1,_ and in addition, the decline in FEV_1_ values was greater and more severe during the 4-year follow-up time, suggesting that TD develops in patients with a more severe disease. Despite this finding, this study [25] did not find a correlation between the Bhalla CT score and the number or size of TD (14.8 ± 3.4 in patients with TD and 12.7 ± 5.0 in patients without TD, *p* = 0.06). TD can be an expression of inflammation or cumulative tracheal stress over time [26]. The lower prevalence can also be due to the different design of this study, since, compared to the study conducted by Gayer et al. [25], we have not actively reviewed the CT images.

We hypothesize that these processes, despite the benefits of ETI therapy, may persist in the presence of *P. aeruginosa* detection. Supporting this hypothesis, the youngest child in our series presented fine tracheal and bronchial granules, suggesting ongoing chronic airway inflammation.

**Figure 6:**
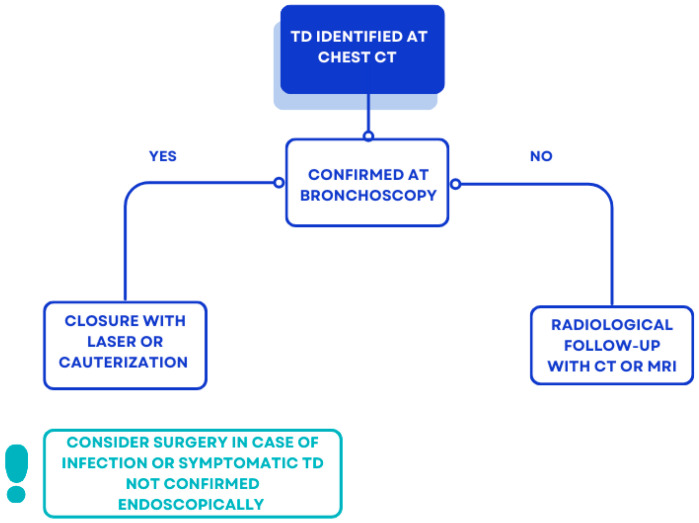
Proposal of management of incidental TD at chest CT in pwCF.

In contrast to the asymptomatic cases we describe, a study of non-CF acquired TD [42], found that a small percentage (11.5%) of patients presented with respiratory symptoms, including hemoptysis, chronic cough, dyspnea, stridor, and recurrent tracheobronchitis. TD can be a reservoir for secretions, causing infection and hemoptysis in some cases [43]. This justifies the usefulness of identifying TD and monitoring their size or any associated symptoms over time, evaluating the need for surgical intervention in selected cases, as reported in Figure 6. Its recognition also allows for the adoption of specific protocols in case of elective intubation to avoid barotrauma during positive pressure ventilation, and avoid causing complications such as tracheal diverticula rupture and subcutaneous emphysema [44].

A previous study [26] reported, in post hoc analyses, an association between hypertonic saline use and a lower incidence of TD, while inhaled antibiotics use was associated with a higher incidence. In our series, when TD was detected, all patients were on inhaled colistimethate, two of them for chronic *P. aeruginosa* colonization, while the youngest for eradication therapy. Twenty-one patients on inhaled colistimethate followed at the CF center of Florence and Naples did not present a TD in the routine chest CT.

CFTR is expressed in the apical membrane of secretory and ciliated cells, where it is implicated in anion and fluid secretion. Ionocytes, mostly present in central airways, have the highest CFTR expression, where it absorbs chloride and fluid [13]. Targeting CFTR with the current modulators improves but still does not alter the viscoelastic characteristics of the mucus in pwCF [45], reducing [46,47], but not restoring the normal level of airway inflammation [45]. Moreover, a recent multicenter observational study from the PROMISE-Micro Study Group [47] found that despite the decreasing of mean sputum densities of bacteria such as *S. aureus* and *P. aeruginosa* after one month of ETI, most patients remained culture-positive; furthermore, those with negative cultures presented still detectable PCR for the pathogens after starting ETI, suggesting the persistence of the infection [48]. Moreover, we should evaluate whether the chest physiotherapy and airway clearance techniques in pwCF undergoing ETI treatment, and leading to a higher endoluminal pressure, play a role in the TD formation. In fact, pwCF in ETI treatment may have been instructed to spend less time conducting their daily physiotherapy [49], and a promising trial [50] will evaluate if exercise can actually replace chest physiotherapy for pwCF under ETI treatment.

In CF, TD may represent a potential source of infection, potentially impacting outcomes, particularly after lung transplantation, although this remains to be definitively established [26]. A study evaluating the role of TD after lung transplantation in pwCF [26] suggested a trend toward increased risk of post-transplant death due to infection, even if not statistically significant, but no differences were found in survival after the lung transplantation, bacterial colonization, and rejection.

A thin section chest CT scan with a 3D reconstruction appears to be the best method to diagnose the TD, since the orifices of the stalks that connect the TD with the tracheal wall may not be endoscopically visible if they are small or fibrous [15,25,26]. Since it has been shown that TDs in CF are usually underreported [22], radiologists should put more stress on TD reporting. MRI can play a role in the diagnosis of TD and information about the tracheal wall as it has higher soft tissue resolution, while it is unable to detect the stalks. MRI has wide intervals between the slices and can fail to detect them [51], as in patient 2. A potential advantage of the MRI compared to CT is in detecting infected TD presenting as paratracheal abscesses and monitor the therapeutic efficacy [51].

Bronchoscopy is usually used to confirm the TD presence, showing a wide-mouthed pouch or even a small pouch filled with secretions [19]. As evidenced in previous studies [25] and confirmed in our case series, bronchoscopy may not be very sensitive in visualizing the TD opening, so the CT appears as the gold standard to assess TD.

If the stalks are not visualized using a CT or bronchoscopy, an upper gastrointestinal endoscopy should be considered [20] to differentiate TD from other conditions such as laryngocele, pharyngocele, Zenker’s diverticulum, apical hernia, and lung bullae [20].

Preventing infection of the TD is of foremost importance. Medical treatment involves prompt antibiotic use in case of infection, and daily chest physiotherapy [19], so usually these conservative managements are already in place for pwCF.

In congenital or symptomatic TD or if the size increases over time, surgical removal with an open neck and a thoracoscopic or thoracotomy approach [19] or an endoscopic approach, such as laser or cauterization, could be considered [15,20].

Follow-ups involve microbiological surveillance to detect and treat any infection and colonization, and imaging with chest CT and MRI. MRI could be preferred in symptomatic patients requiring conservative close observation and medical management since it can sensitively reveal changes and guide the treatment plan [51]. We suggest following up with the lesion with a bronchoscopy in case the stalk is endoscopically evident, or radiologically in case the patient continues to be asymptomatic, and if no orifice is visible during the bronchoscopy.

## 5. Conclusions

To our knowledge, this is the first case series describing the presence of TD in patients with CF in treatment with ETI.

The study could suggest that despite ETI treatment, chronic airway inflammation, also due to the persistence of *P. aeruginosa* detection, could still not be completely addressed, and could potentially lead to TD, thus being as a marker of persistent inflammation.

However, these patients could have formed TD prior to initiating ETI, since the previous imaging had not been performed prior of the start of the ETI.

The main limits of this study are due to the retrospective design of the study and the relatively small numbers of cases.

Future studies are needed to evaluate the clinical course of TD in pwCF to guide the follow-up and the possible association between airway infection, inhaled antibiotics, and the presence of TD, as well to define the best prevention and treatment options.

## Figures and Tables

**Table 1:** Clinical presentation of tracheal diverticula in described people with Cystic Fibrosis.

Age (Years), Sex	*CFTR* Genetic Profile	Chronic Colonization	ppFEV_1_ (%)	ETI Months	TD n, Size (mm) and Position	Confirmed by Bronchoscopy	Communication with the Trachea	Follow-Up
30, M	F508del/ E585X	SA, PA	73	36	2, 10 mm, right posterolateral at T2 level	Yes	Visible radiologically and endoscopically	Bronchoscopy after 6 months: stable TD; occurrence of punctiform paracarenal TD
13, F	F508del/ Delexon22–24	SA, PA	63	12	1, right paratracheal at T2-T3 level	No	Not identified	MRI after 6 months: TD non reported
10, F	F508del/ F508del	SA, first PA detection	117	16	1, 9, right posterolateral at T1 level	No	Visible radiologically, not endoscopically	Not yet performed

## Data Availability

Data sharing is not applicable to this article as no datasets were generated or analyzed during the current study.

## Abbreviations

CF	Cystic Fibrosis
CFTR	cystic fibrosis transmembrane conductance regulator
CT	chest computerized tomography
EMA	European Medicines Agency
ETI	Elexacaftor/Tezacaftor/Ivacaftor
FDA	Food and Drug Administration
MRI	Magnetic Resonance Imaging
PwCF	People with cystic fibrosis
SC	Sweat chloride
TD	tracheal diverticula
